# Methylomic changes in individuals with psychosis, prenatally exposed to endocrine disrupting compounds: Lessons from diethylstilbestrol

**DOI:** 10.1371/journal.pone.0174783

**Published:** 2017-04-13

**Authors:** Fabrice Rivollier, Boris Chaumette, Narjes Bendjemaa, Mélanie Chayet, Bruno Millet, Nematollah Jaafari, Amina Barhdadi, Louis-Philippe Lemieux Perreault, Sylvie Provost, Marie-Pierre Dubé, Raphaël Gaillard, Marie-Odile Krebs, Oussama Kebir

**Affiliations:** 1 Université Paris Descartes, Université Paris Sorbonne Paris Cité, Centre de Psychiatrie et Neurosciences, UMR S 894, Paris, France; 2 INSERM, Laboratoire de Physiopathologie des Maladies Psychiatriques, Centre de Psychiatrie et Neurosciences, UMR S 894, Paris, France; 3 CNRS, GDR3557-Institut de Psychiatrie, Paris, France; 4 Faculté de Médecine Paris Descartes, Centre Hospitalier Sainte-Anne, Service Hospitalo-Universitaire, Paris, France; 5 Department of Adults Psychiatry, ICM-A-IHU, UPMC UMR S 975, Inserm U 1127, CNRS UMR 7225, GH Pitié-Salpêtrière, Paris, France; 6 Unité de Recherche Clinique en Psychiatrie Pierre Deniker, Centre Hospitalier Henri Laborit, INSERM CIC-P 1402, INSERM U 1084 Laboratoire Expérimental et Clinique en Neurosciences, Univ Poitiers, CHU Poitiers, Groupement De Recherche CNRS 3557, Poitiers, France; 7 Université de Montréal, Beaulieu-Saucier Pharmacogenomics Center, Montréal Heart Institute, Montréal, QC, Canada; Meharry Medical College, UNITED STATES

## Abstract

**Background:**

In the Western world, between 1940 and 1970, more than 2 million people were exposed *in utero* to diethylstilbestrol (DES). In exposed individuals, and in their descendants, adverse outcomes have been linked to such exposure, including cancers, genital malformations, and less consistently, psychiatric disorders. We aimed to explore whether prenatal DES exposure would be associated with DNA methylation changes, and whether these epigenetic modifications would be associated with increased risk of psychosis.

**Methods:**

From 247 individuals born from mothers exposed to DES, we selected 69 siblings from 30 families. In each family, at least one sibling was exposed in utero to DES. We performed a methylome-wide association study using HumanMethylation450 DNA Analysis BeadChip® in peripheral blood. We analyzed methylation changes at individual CpGs or regions in exposed (n = 37) versus unexposed individuals (n = 32). We also compared exposed individuals with (n = 7) and without psychosis (n = 30).

**Results:**

There were more individuals with schizophrenia in the DES-exposed group. We found no significant differences between exposed and unexposed individuals with respect to differentially methylated CpGs or regions. The largest difference was in a region near the promoter of an ADAMTS proteoglycanase gene (*ADAMTS9*). Compared to exposed individuals without psychosis, exposed individuals with psychosis had differential methylation in the region encompassing the gene encoding the zinc finger protein 57 (*ZFP57*).

**Conclusions:**

*In utero* exposure to DES was not associated with methylation changes at specific CpG or regions. In exposed individuals, however, psychosis was associated with specific methylomic modifications that could impact neurodevelopment and neuroplasticity.

## Introduction

Between 1940 and 1970 in the Western world, about 2 million pregnant women received diethylstilbestrol (DES), a synthetic estrogen prescribed to prevent complications during pregnancy [1]. In the late 1960s, an unusual cluster of vagina and cervix anomalies were described in daughters of women treated with DES [2]. An increased risk of uterine malformations and vaginal clear cell adenocarcinoma was later confirmed by the longitudinal follow up of cohorts of prenatally DES-exposed women [3]. In sons of DES treated mothers, an increased risk of urogenital malformations was also suggested [4]. More recently, evidence for a trans-generational transmission of this risk to grandsons was observed [5–7]. Furthermore, although inconsistently reported, increased risk for complex conditions, such as immunological diseases and psychiatric disorders, may also exist in prenatally exposed individuals [8]. The exact mechanism for how DES increases the risk for these various complex conditions is unknown, yet disruption of epigenetic homeostasis has been proposed as a tentative hypothesis [9,10]. Many animal studies have identified DES-exposure related epigenetic modifications, including DNA methylation changes [9–12], while the results of human studies remain inconclusive [13].

Exposure to DES in humans can be considered a rare opportunity for investigating the effects of time-limited, exposure to an endocrine disruptor during a highly vulnerable window. Indeed, hormonal regulation during the perinatal period strongly impacts behavioral development of the offspring [14–16]. More specifically, prenatal exposure to DES has been shown to alter mating behavior in female rats [17] and guinea pigs [18] (lower lordosis quotient with more rejections to male mounting behavior compared to controls) and to increase aggression towards conspecifics in both female and male mice [19]. Compelling evidence also demonstrates that early brain development disruption can result in increased psychiatric or behavioral alterations in adulthood [20]. Among psychiatric disorders, it has been suggested that epigenetic modifications may specially influence the emergence of psychotic disorders [21–24]. These observations raise the possibility that DES may increase the risk of psychiatric disorders in prenatally exposed individuals, although evidence supporting this hypothesis has been scarce [8].

We hypothesized that prenatal DES exposure would be associated with persistent DNA methylation changes detectable in peripheral cells and that these epigenetic modifications would be involved with increasing the risk of psychosis. We conducted a study of pangenomic DNA methylation patterns in whole blood from individuals born from mothers who reported use of DES during pregnancy, and compared them to their non-exposed siblings. Among exposed individuals, we further investigated differentially methylated regions in a small number of individuals with psychosis.

## Methods and materials

### Study participants

Our study was promoted by Inserm (Institut National de la Santé et de la Recherche Médicale) and approved by the institutional ethics committee “Comité de protection des personnes, Ile-de-France 4, Paris, France”, and followed Helsinki’s declaration of human rights. Written informed consent was obtained from all participants prior to their participation.

Individuals (n = 247) from families with DES exposure history were recruited through a users’ association (Hhorages, Halte aux HORmones Artificielles pour les GrossessES). The goal of the association was to assess a causal relationship between exposure to diethylstilbestrol and long-term side effects, with a focus on psychiatric effects. From these 247 patients, we selected 75 siblings from families where at least one sibling was exposed *in utero* to DES or ethynil estradiol (n = 3 individuals), the other siblings were either exposed or unexposed. Mothers were interviewed and provided with a list of all the brand names of DES and/or ethynil estradiol medicines marketed in France and Europe between 1940–1970. Medical records, medical prescriptions and children health record were obtained and examined. We excluded all individuals for whom the exposure to DES was doubtful. Indeed, memory bias was largely avoided in our study, because a majority of the exposures were documented by prescriptions or clinical records. In total, 41 exposed and 34 unexposed siblings from 31 families were selected. The families were not related between each other.

Each individual was interviewed face-to-face by a trained psychologist. Exposure characterization, obstetrical and medical history, demographic and phenotypic data were collected through standardized questionnaires (i.e. the Pedigree and the Familial Interview for Genetic Studies with additional standardized questionnaire for exposure to medication and obstetrical complications, Diagnostic Interview for Genetic Studies (version 3.0, French translation, validated scales for psychopathology, data not shown). Diagnoses were performed according to DSM-IV-TR criteria.

Blood samples were collected from those 75 subjects and frozen within the hour. For each individual, genomic DNA (500 ng) was extracted from whole blood and treated with sodium bisulfite, using the EZ-96DNA Methylation KIT (Catalog No D5004, Zymo Research, USA), following the manufacturer’s standard protocol. Then, methylation profiles were assessed using HumanMethylation450 DNA Analysis BeadChip® (Illumina, San Diego, CA, USA). This technology allows the study of the methylation state of 485 577 CpGs throughout the genome. The CpGs site are region of DNA where multiple cytosine-guanine pairs are clustered together upstream of a gene, in the regulatory region of that gene. Cytosines in CpGs can be methylated, changing the gene expression. No batch effect was detected. R packages methylumi and watermelon were used for data quality check and normalization. Computations and statistical analyses related to quality control can be found in the supplementary appendix **(S1 Appendix).** Steps used for data clean-up procedure and normalization comprised gender check between phenotype file and methylation data set. Subsequent clean-up states comprised flagging and removing individuals with no result or gender discrepancies or discordance genotypes, samples with ≥ 1% of sites with a detection P-value ≥ 0,05, probes with beadcount < 3 in ≥ 5% of samples, probes with ≥ 1% of samples with a detection P-value ≥ 0,0. Additionally, probes on chromosome X and Y, single-nucleotide polymorphism probes at the CpG site and non–specific probes that map to more than one location in the genome were removed. After quality control, six samples were removed. After normalization and data clean-up, analyses were finally conducted in 69 individuals (n = 37 exposed, n = 32 unexposed) using 411 947 CpG.

### Genomic methylation analyses

#### Methylome-wide association study (MWAS)

We used a classic methylome-wide association analysis, comparing exposed and unexposed individuals for methylation changes at each CpG site. We used a linear model with moderated t-statistic and controlled for potential confounding factors such as age, gender, psychosis, family and genital abnormalities. The multiple-testing-adjusted significance threshold for probe-wise analysis was established at P = 1.2 x 10^−7^ (0.05/411,947 analyzed probes). Analyses were conducted using the R (version 3.1.0) limma package.

#### Differentially methylated regions (DMRs)

We then used the *bumphunter* algorithm (from the R minfi package) to look for associations between beta-values of genomic regions and phenotypes. We used the following parameters: methylomic cut-off = 5%, nullMethod = bootstrap and 1000 permutations. In the context of the 450k array, the algorithm first defines clusters of CpG. Clusters are groups of probes such that two consecutive probes locations in the cluster are not separated by more than short range distances, as supplied by the manufacturer. The method based on DMRs was used to determine associations with prenatal exposure to DES, corrected by age, gender, psychosis and cell counts. Furthermore, considering that DES exposure could hypothetically have different consequences in individuals we examined specific methylation differences between exposed individuals with psychosis and exposed siblings without psychosis. Top significant findings were selected with the following criteria: (Corrected p-value <0,05, length > 1 base, excluding region including HLA-DQ and HLA-DRB, chromosome 6 [32523136; 32633163], because of frequent recurrence of this finding by minfi package suggesting spurious results due to the algorithm (according to its authors). Multiple testing correction have been performed following to approaching: Family-wise error rate (FWER) and Bonferroni correction. FWER is given by the proportion of bootstrapped results for which any region was as or more extreme as the observed results for the region under consideration. Bonferroni correction is calculated by multiplication of the nominal p-value by the total number of tested DMRs. Genomic annotation was done according to hg19 release.

## Results

### Clinical description (Table 1)

Analyses were conducted in 69 individuals, 37 exposed (12 males and 25 females) and 32 unexposed (12 males and 20 females). The exposed and unexposed siblings were comparable in terms of gender and age. Psychiatric diseases were highly prevalent in both groups, but there were significantly more psychiatric conditions in exposed individuals compared to non-exposed participants. The prevalences of depression, bipolar disorder or anxiety disorder were similar between groups. However, psychosis, and especially schizophrenia, were more prevalent in the exposed siblings: 7 patients (6.7%) met the diagnostic criteria for schizophrenia (n = 4) or schizoaffective disorder (n = 3) vs none in the unexposed group (p = 0.009). A detailed description of all 69 patients can be found in **supplementary Table 1 (S1 Table)**.

**Table 1:** Hazard Ratios for Psychiatric Phenotypes in Siblings with and Those without Diethylstilbestrol Exposure.

	Exposed n = 37	Unexposed n = 32	p
Male sex—n (%)	12 (32,4%)	12 (37,5%)	0,551
Age–(year)	41,2	42,2	0,511
Years of studies	13,46	13,94	0,513
Schizophrenia and schizoaffective disorder–n (%)	7 (6,7%)	0 (0,0%)	0,009
Depression—n (%)	19 (51,4%)	13 (40,6%)	0,373
Bipolarity–no (%)	3 (8,1%)	2 (6,3%)	0,767
Anxiety disorder—n (%)	7 (18,9%)	3 (9,4%)	0,261
Any psychiatric diseases—n (%)*	35 (94,6%)	22 (68,8%)	0,005

* Any psychiatric diseases includes schizophrenia spectrum and other psychotic disorders, bipolar and related disorders, depressive disorders, anxiety disorders, obsessive-compulsive and related disorders, as well as feeding and eating disorders.

### Methylome-wide association study (MWAS)

Methylation changes at specific CpG loci (Differentially Methylated Positions, DMP) associated with exposure to DES were investigated. None of these loci reached significance at a genome-wide level. QQ-plot, manhattan plot and best 100 results are displayed in **supplementary material (S1 Fig, S2 Fig, S2 Table)**.

### Differentially methylated regions (DMRs)

1. **DMRs associated with prenatal exposure to DES (Table 2):** 1089 DMRs with an inter-group difference reaching more than 5% of methylation rate were further considered in the analyses. None of the DMRs reached statistical significance after FWER correction. Top finding was a cluster of 2 CpGs located in a CpG island in chromosome 3 (64,670,013–64,670,017) near the promoter of the gene encoding the A Disintegrin-Like And Metalloprotease (Reprolysin Type) With Thrombospondin Type 1 Motif, 9 (*ADAMTS9*) (p = 0.01, FWER = 0.8). This cluster displayed a 12% difference in methylation between the two groups of siblings, with less methylation in the exposed group.
2. **DMRs associated with psychosis (Table 3):** 7413 DMRs overpassed the inter-group methylation difference threshold and were further considered in the analyses. One DMR reached statistical significance: a cluster of 25 CpGs located in chromosome 6 (29,648,161–29,649,092) in the zinc finger protein 57 gene (*ZFP57*). The cluster displayed a 15% difference in methylation (p = 6,6.10^−06^; corrected p-value <0,05), with more methylation in the psychosis group.

**Table 2:** DMRs associated with prenatal exposure to DES. Top findings (fwer < 0,8, length > 1 base, HLA-DQ and HLA-DRB excluded).

Chr	start	end	genes	p-value	fwer	Bonferroni
Chr3	64670013	64670017	*ADAMTS9*	0,01131	0,796	1

**Table 3:** DMRs associated with psychosis in exposed individuals. Top findings (fwer < 0,8, length > 1 base, HLA-DQ and HLA-DRB excluded).

Chr	start	end	genes	p.value	fwer	Bonferroni
Chr6	29648161	29649092	*ZFP57*	6.581e-06	0.052	0.048
Chr19	12876846	12877188	*HOOK2*	7.340e-06	0.054	0.054
Chr6	32604564	32604865		0.0002162	0.648	1
Chr10	123244536	123244591	*FGFR2*	0.0002368	0.67	1
Chr10	123355268	123356041	*FGFR2*	0.0001936	0.676	1
Chr6	32557970	32558459		0.0002564	0.707	1
Chr17	79905236	79905263		0.0002689	0.726	1
Chr22	24384245	24384525	*GSTT1*	0.0001884	0.756	1
Chr6	291687	293331	*DUSP22*	0.0001831	0.771	1
ChrX	8751190	8751687		0.0002200	0.78	1

## Discussion

DES, a synthetic estrogen and potent endocrine disruptor, was prescribed during pregnancy to avoid miscarriage. DES exposure thus represents a unique model of high-dose prenatal exposure to an endocrine disruptor in humans. It is associated with a wide range of medical consequences, including cancer and genital anomalies, such as adenocarcinoma of the vagina [2–4]. Aside from DES, exposure to several endocrine disrupting compounds have been associated with neurodevelopmental consequences. In rats, chlorpyfiros (CPF) exposure alters neuronal differentiation, synaptogenesis and serotoninergic and dopaminergic transmission [25]. In human, exposure to polychlorinated biphenyl (PCB) has been associated with alteration of cognitive functions [26]. Behaviors alterations has been associated as well in humans (exposure to Bisphenol A [27] and PCBs [28]) and in rats (exposure to CPF [29] and bisphenol A [30,31]).

Animal studies have demonstrated an association between exposure to endocrine disruptors and epigenetic modifications that are thought to be damaging [8, 9, 11, 24–26]. Yet, the precise molecular mechanism in humans is unclear. We hypothesized that persistent DNA methylation changes might be detectable in the blood of Caucasian individuals exposed prenatally to DES compared to non-exposed siblings. Our analysis did not find specific significant differences, at CpG level, in methylation between the two groups. This negative result could be due to heterogeneity of methylomic changes induced by DES leading to difficulties in identifying statistical signal.

Harlid et al. recently published the first study to evaluate possible effects of DES exposure on genome-wide DNA methylation in humans [13]. They studied whole blood DNA methylation in one hundred 40–59 years old women reporting *in utero* exposure, compared to 100 unexposed women, belonging to a large cohort of women with a family history of breast cancer. The authors used a genome-wide approach and they focused on a subset of candidate genes known to have altered expression in mice uterine or vaginal tissue following pre- or perinatal- exposure to DES. They did not found any CpGs with a genome-wide significance, but the DMR approach was not used.

In the present study, the most significant DMR associated with DES exposure was found near the promoter of *ADAMTS9* gene. ADAM-TS/metallospondin genes encode a family of proteins with structural homology to the ADAM metalloprotease-disintegrin family [32]. Members of the ADAM-TS family have been implicated in the control of organ shape during development [33], most notably to the development and function of the uterus and reproductive organs [34,35] which can be abnormal after intra-utero DES exposure. A significant correlation between *ADAMTS9* methylation and loss of expression of ADAMTS9 was observed in gastric, colorectal and pancreatic cancers by real-time PCR [36]. Physiologically, ADAMTS proteoglycanases are synthesized mainly by cerebral astrocytes and expressed in several CNS structures, including cortex, hippocampus, striatum and spinal cord. Increasing evidence suggests that they may play critical roles in the control of the CNS development. Indeed, several functions in the physiological and pathological CNS, such as neuroplasticity, neurorepair, inflammation and angiogenesis, may be achieved via the cleavage of their substrate (chondroitin sulfate proteoglycans or Reelin), but also independently of their proteolytic activity [37]. Interestingly, these developmental effects overlap with side effects that have already been reported in relation to DES exposure. Indeed, DES was found to significantly modulate long-term potentiation and synaptic plasticity in the hippocampus in rats [38,39].

One DMR located in the *ZPF57* gene was significantly more methylated in exposed individuals with psychotic disorders compared to exposed individuals without psychosis. *ZPF57* is a Kruppel-associated box (KRAB) preferentially expressed early in development [40]. It is a transcriptional regulator, acting in the maintenance of imprinting [26,41] and in DNA methylation [42,43]. It has been described as a folate-sensitive region during *in utero* development [44]. More specifically, SNPs in *ZFP57* have been associated with the immune response [45]. High grade glioblastoma has also been associated with aberrant *ZFP57* expression [46]. In a recent study, Ladd-Acosta et al. identified a difference in methylation between autistic and control females in cerebellar tissue, with *ZFP57* being more methylated in autistic patients [47]. Autism and schizophrenia are both linked to deviant neurodevelopment and plasticity, and they have shared features [48,49]. Overall, our results suggest that in exposed individuals, *ZFP57* methylation may be associated with psychosis. This specific differential methylation, which affects transcriptional regulation and DNA methylation mechanisms, may therefore have a broad impact on transcription of numerous genes, thus leading to heterogeneous consequences, especially during neurodevelopment.

How can we interpret these methylomic changes supposedly associated with prenatal exposure to DES? According to the “signature model”, DES exposure could leave a methylomic signature in blood, without any causal relation to the epigenetic modifications or pathophysiological mechanisms associated with phenotype outcomes. DES acts as an estrogenic compound and could, during development, modify a wide range of tissues, depending on their vulnerability window. Thus, the blood signature could be a possible biomarker of DES exposure and/or of the emergence of disorders beyond the disruption of a specific tissue. In contrast, the “functional mirror model” suggests that the methylation status of a site in the blood is correlated with a corresponding site in other organs, such as the uterus or brain, which may be of relevance for the disease’s etiology [50]. Methylation changes in blood would only mirror the ones occurring in damaged tissues. For example, recent studies described a relative concordance between methylation profiles in the brain and blood [51,52]. Nevertheless the magnitude of methylation levels could be inferior in the blood compared to those present in more central tissues [53].

Our study has several strengths. First, all of the patients were interviewed face-to-face and diagnoses were done by trained psychologist according to standardized questionnaires. Then, we compared exposed subjects to their unexposed siblings, who shared environmental and genetic factors that could influence DNA methylation. Last, we used a DMR analysis, a recent technique proposed to produce results with higher biological relevance than those obtained by MWAS based on single points.

Limitations of our study should be noted. First, the relatively small number of subjects included contributed to a lack of statistical power. Patients exposed to DES are not easy to recruit and proof of their exposure is often difficult to obtain. However, memory bias was largely prevented in our study, because a majority of exposures were documented by prescriptions or clinical records. This stringent criterion resulted in a reduced sample size. Moreover, due to the fact that the exposure was around fifty years ago, there were not enough available data in order to evaluate what dose was received, for how long, and at what time during the pregnancy. These data would have been valuable. Second, we recruited patients through a users’ association, which could have contributed to selection bias. Indeed, there was an overrepresentation of psychiatric diseases in these participants when compared to the general exposed population. However, there was no previous hint that the potential bias could influence the biological substratum of gene-environment-epigenome complex interactions. Finally, the HumanMethylation450 DNA Analysis BeadChip® chip allows a coverage 10 time less dense than methylation sequencing (Meth-seq). Thus, there is a possibility that epigenetic modifications related to DES exposure were not detectable by our method.

Our study raises the question of in utero exposure to endocrine disruptors, including those found the environment, and its impact on neurodevelopment through epigenetic mechanisms. Our specific population being scarce, future directions may focuse on other endocrine disruptors. In DES-exposed patients, as well as for other endocrine disruptors, new results raise the question of a transgenerational effect. This may lead to concern about endocrine disruptors exposure, and make future researches in grandchildren, even more relevant.

## Conclusion

We hypothesized that persistent DNA methylation changes might be detectable in the blood of individuals exposed prenatally to DES. Our analyses in Caucasian siblings did not find conclusive evidence of methylation changes associated with DES exposure. Yet, we report that within the exposed population, increased methylation in the region encompassing the *ZPF57* gene was significantly associated in a small number of patients with psychotic disorders. This result is in line with the observed changes in *ZPF57* methylation that were seen in post-mortem brain tissue from individuals with autism. Further investigations are needed to explore more precisely the role of *ZPF57* in psychotic disorders and schizophrenia. Beyond DES, environmental endocrine disruptors are numerous and highly prevalent (e.g. bisphenol A) but their developmental consequences are largely unknown and further studies are warranted in order to better understand the epigenetic changes induced by these environmental endocrine disruptors.

## Supporting information

S1 Appendix Computations and statistical analyses related to quality control. (DOCX)

S1 Fig QQ-Plot Regarding Methylation changes at specific CpG loci (Differentially Methylated Positions, DMP) associated with exposure to DES. (DOCX)

S2 Fig Manhattan Plot Regarding Methylation changes at specific CpG loci (Differentially Methylated Positions, DMP) associated with exposure to DES. (DOCX)

S1 Table Clinical description of all 69 patients. (XLSX)

S2 Table Top 100 CpG loci associated with exposure to DES are investigated. (DOCX)
