# Supplementary material for: Methylomic changes in individuals with psychosis, prenatally exposed to endocrine disrupting compounds: Lessons from diethylstilbestrol
Source: PLoS One. 2017 Apr 13;12(4):e0174783. doi: 10.1371/journal.pone.0174783 (PMC5390994; doi:10.1371/journal.pone.0174783)
Supplement: S1 Appendix — (DOCX) [file pone.0174783.s001.docx]

**Supplementary Appendix**: Computations and statistical analyses related to quality control.

Quality Control: Meth450K used two kinds of probes: probes for methylated loci and probes for unmethylated loci. The ratio between these probes was used as a metric for classifying low quality samples, which were removed from the subsequent analyses.


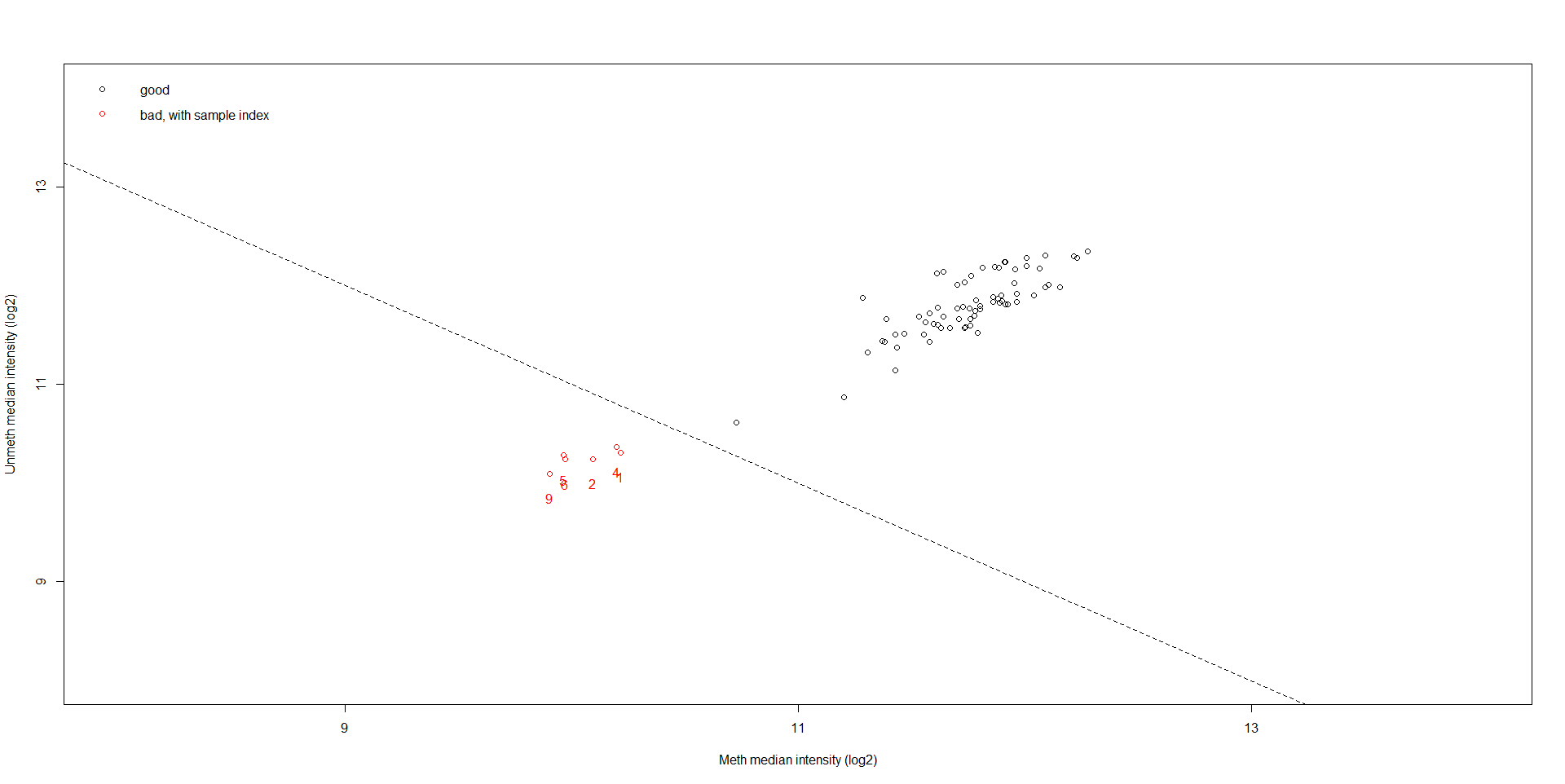


Density Plot: The density given by Minfi package plot represents the number of probes (CpG) for each level of methylation (beta-value).
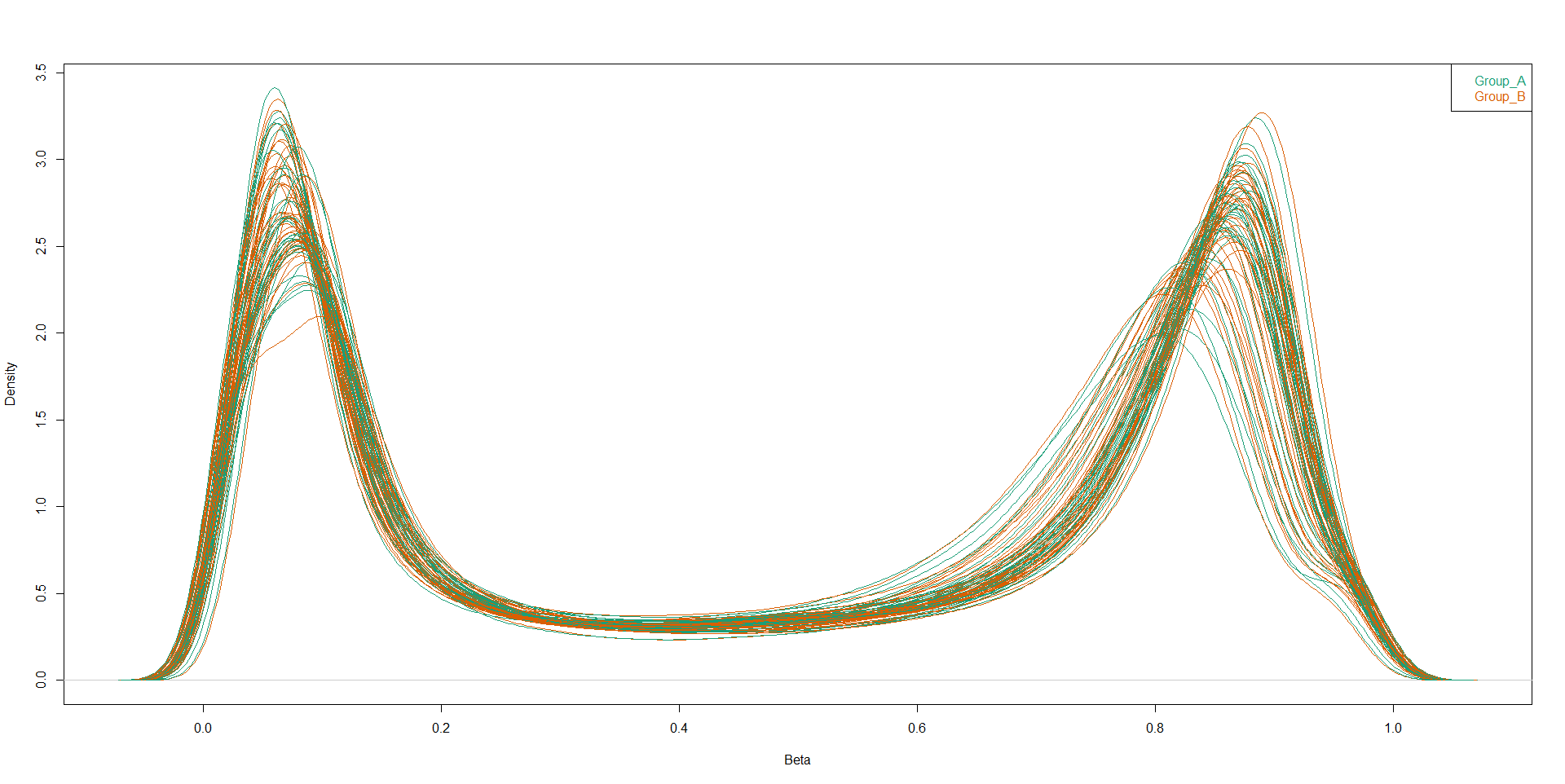


Summary information about data clean-up procedure

|  |  | sites |  |
| --- | --- | --- | --- |
| Total number of methylation sites | 485 577 |  |  |
| Total number of samples | 78 |  |  |
| Samples having 1% of sites with a detection p-value greater than 0.05 removed | 0 |  |  |
| Sites with beadcount <3 in 5% of samples removed | 438 | -4,750^a^ |  |
| Sites having 1% of samples with a detection p-value greater than 0.05 removed | 4455 |  |  |
| Non-specific sites removed* | 41 937 | -57,631^b^ |  |
| Sites with SNPs within the CpG probes removed * | 20 869 |  |  |
| Probes on X and Y chromosomes removed | 11 184 | -11,184 |  |
| SNP probes | 65 | -65 |  |
| **total number of sites** | **411 947** |  |  |
| **total number of samples** | **78** |  |  |

***** from Price *et al* (11)

^a^ 4,750 unique probes were removed (143 present in both categories)

^b^ 57,631 unique probes were removed (1,279 were also in the sites having 1% of samples with a detection p-value greater than 0.05 removed and 1,362 were on X or Y chromosomes (60,272 unique probes -1362 -1279= 57,631)
