# Supplementary material for: Methylomic changes in individuals with psychosis, prenatally exposed to endocrine disrupting compounds: Lessons from diethylstilbestrol
Source: PLoS One. 2017 Apr 13;12(4):e0174783. doi: 10.1371/journal.pone.0174783 (PMC5390994; doi:10.1371/journal.pone.0174783)

**Supplementary Figure 2**: Manhattan Plot Regarding Methylation changes at specific CpG loci (Differentially Methylated Positions, DMP) associated with exposure to DES.
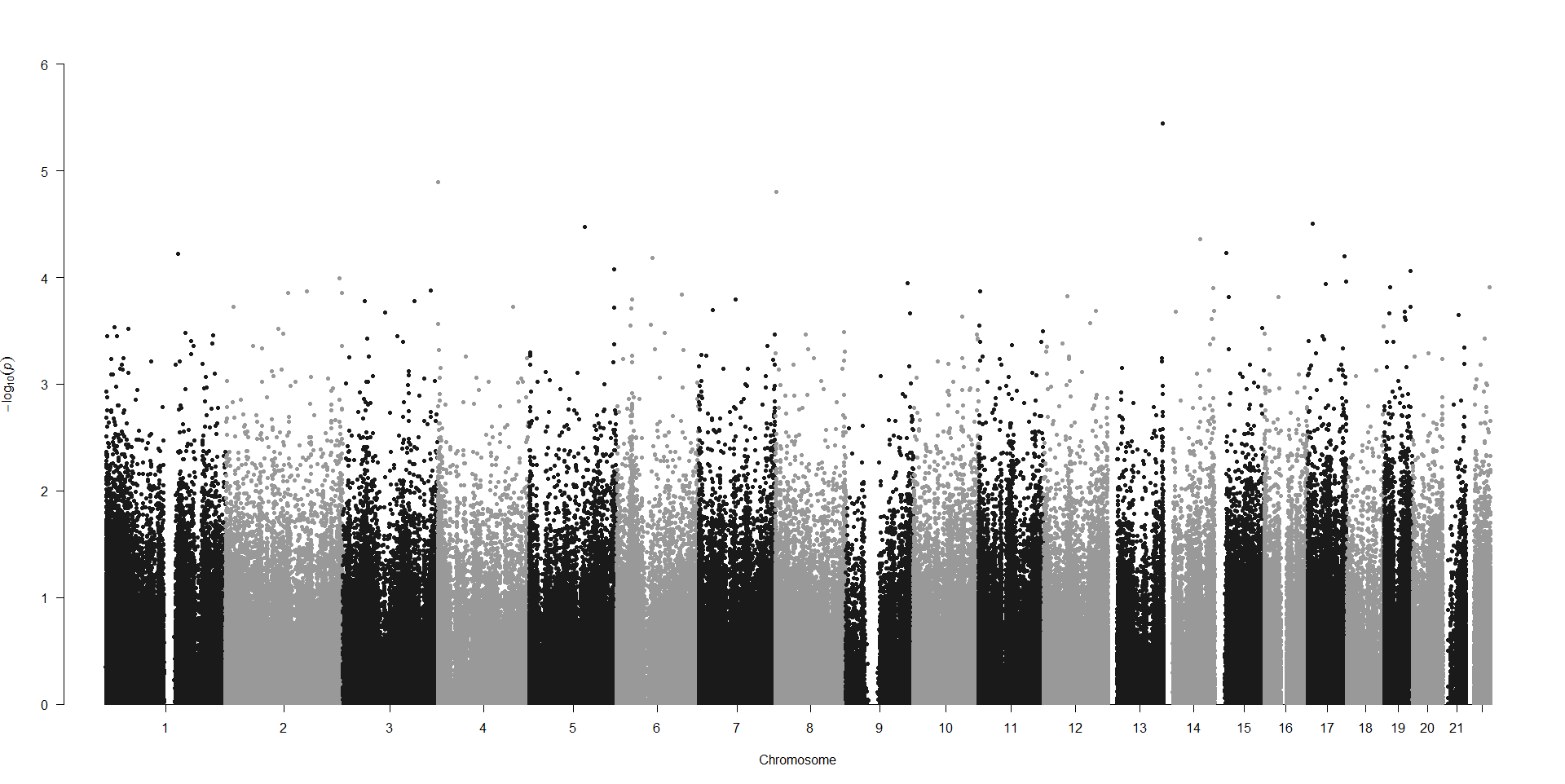

Supplement: S2 Fig — (DOCX) [file pone.0174783.s003.docx]
