# Supplementary material for: Methylomic changes in individuals with psychosis, prenatally exposed to endocrine disrupting compounds: Lessons from diethylstilbestrol
Source: PLoS One. 2017 Apr 13;12(4):e0174783. doi: 10.1371/journal.pone.0174783 (PMC5390994; doi:10.1371/journal.pone.0174783)
Supplement: S2 Table — (DOCX) [file pone.0174783.s005.docx]

**Supplementary Table 2**: Top 100 CpG loci associated with exposure to DES are investigated.
